# Whole brain surface-based morphometry and tract-based spatial statistics in migraine with aura patients: difference between pure visual and complex auras

**DOI:** 10.3389/fnhum.2023.1146302

**Published:** 2023-04-18

**Authors:** Chiara Abagnale, Antonio Di Renzo, Gabriele Sebastianelli, Francesco Casillo, Emanuele Tinelli, Giada Giuliani, Maria Giulia Tullo, Mariano Serrao, Vincenzo Parisi, Marco Fiorelli, Francesca Caramia, Jean Schoenen, Vittorio Di Piero, Gianluca Coppola

**Affiliations:** ^1^Department of Medico-Surgical Sciences and Biotechnologies, Sapienza University of Rome Polo Pontino ICOT, Latina, Italy; ^2^IRCCS–Fondazione Bietti, Rome, Italy; ^3^Unit of Neuroradiology, Department of Medical and Surgical Sciences, Magna Græcia University, Catanzaro, Italy; ^4^Department of Human Neurosciences, Sapienza University of Rome, Rome, Italy; ^5^Headache Research Unit, Department of Neurology, CHU de Liège, Citadelle Hospital, Liège, Belgium

**Keywords:** surface-based morphometry (SBM), tract-based spatial statistics (TBSS), migraine aura, lingual gyrus, Rolandic operculum

## Abstract

**Background:**

The migrainous aura has different clinical phenotypes. While the various clinical differences are well-described, little is known about their neurophysiological underpinnings. To elucidate the latter, we compared white matter fiber bundles and gray matter cortical thickness between healthy controls (HC), patients with pure visual auras (MA) and patients with complex neurological auras (MA+).

**Methods:**

3T MRI data were collected between attacks from 20 patients with MA and 15 with MA+, and compared with those from 19 HCs. We analyzed white matter fiber bundles using tract-based spatial statistics (TBSS) of diffusion tensor imaging (DTI) and cortical thickness with surface-based morphometry of structural MRI data.

**Results:**

Tract-based spatial statistics showed no significant difference in diffusivity maps between the three subject groups. As compared to HCs, both MA and MA+ patients had significant cortical thinning in temporal, frontal, insular, postcentral, primary and associative visual areas. In the MA group, the right high-level visual-information-processing areas, including lingual gyrus, and the Rolandic operculum were thicker than in HCs, while in the MA+ group they were thinner.

**Discussion:**

These findings show that migraine with aura is associated with cortical thinning in multiple cortical areas and that the clinical heterogeneity of the aura is reflected by opposite thickness changes in high-level visual-information-processing, sensorimotor and language areas.

## 1. Introduction

The migraine aura is a neurological phenomenon that may precede and accompany the headache phase of the migraine attack. In the majority of cases (around 90%), aura symptoms are visual, consisting of scintillating scotomas or fortification spectra (Rasmussen and Olesen, 1992). In a minority of patients, the aura also comprises somatosensory disturbances, such as paresthesia usually with a cheiro-oral distribution, and less frequently language symptoms (ICHD, 2018). Of note, when aura symptoms are multiple, they generally follow one another in a stereotyped sequence that begins with visual symptoms, then proceeds to sensory symptoms and finally to language disturbances. Systematic studies (Rasmussen and Olesen, 1992) have demonstrated that patients with sensory and/or language symptoms almost always experience visual symptoms; conversely, patients with visual aura develop sensory and/or language disturbances only occasionally. The most likely pathophysiological phenomenon causing the aura symptoms is cortical spreading depression (Leão, 1944).

We have previously hypothesized that the heterogeneity of the aura phenotype symptoms might be associated with differences in brain function and anatomy, specifically between migraine patients with pure visual auras (MA) and those with complex neurological auras, defined as visual aura plus at least one of sensory and language symptoms (Ambrosini et al., 1999; Coppola et al., 2015). Both electrophysiological (Ambrosini et al., 1999; Coppola et al., 2015) and neuroimaging studies (Sándor et al., 2005; Petrusic et al., 2019; Coppola et al., 2021; Silvestro et al., 2022) support this hypothesis. More recent studies also show abnormalities in connectivity between various brain networks, in particular default mode and dorsal attention networks, or in DTI metrics in the thalamus (Coppola et al., 2021) as well as insula and lingual gyrus in patients with complex auras compared to those with pure visual auras (Silvestro et al., 2022).

Taken together these data point toward an extensive, but different cortical involvement depending on the aura phenotype. This is further supported by the finding that thickness of the visual and somatosensory cortices differs between patients with pure visual or complex auras, although these patients were not compared with healthy subjects (Petrusic et al., 2019). Whether these morphometric abnormalities may be a consequence of alterations in white matter fiber bundles (Granziera et al., 2006, 2014; DaSilva et al., 2007; Rocca et al., 2008; Szabó et al., 2018; Faragó et al., 2019) or caused by strictly cortical phenomena remains to be clarified. We decided therefore to analyze with 3T MRI in the same patients both cerebral white matter fiber bundles using a tract-based spatial statistics (TBSS) analysis of diffusion tensor imaging (DTI) and cortical thickness using surface-based morphometry. Based on the abovementioned studies, we hypothesized that patients with complex auras have more pronounced anatomical brain alterations than those with pure visual auras and healthy subjects.

## 2. Materials and methods

### 2.1. Participants

This study is part of a larger research program in which the same patients underwent different MRI acquisitions during the same scanning session. Some of the acquired data, analyzed with other methods, have been published elsewhere (Coppola et al., 2021). We scanned 40 patients suffering from migraine with typical aura (ICHD-III code 1.2.1) who were referred to the headache center of the Policlinico Umberto I (Sapienza University of Rome) or to the Polo Pontino headache center (Sapienza University of Rome) in Latina.

Patients with brainstem aura, hemiplegic aura, persistent aura without infarction or evidence of a brain lesion on structural MRI were excluded, as were patients who were taking prophylactic migraine treatments or had taken one in the 3 months preceding the study. All patients were recorded during the interictal period, at least 3 days before and after an attack, which was ensured by collecting their headache diaries and a telephone interview after the scanning.

Exclusion criteria for all study participants included having a history of another neurological or psychiatric disorder, as well as having autoimmune, endocrinological, connective tissue, chronic extracerebral painful, or neuro-ophthalmological diseases, as determined by a thorough neuro-ophthalmological examination that included a visual acuity test, an intraocular pressure measurement, and indirect ophthalmoscopy.

All patients enrolled in the study had to fill in a headache diary that was mailed to them at least 3 months before the first visit, We collected the following clinical information from the patients’ diaries: attack frequency (n/month), duration of migraine history (years), mean severity of migraine attacks [0–10 on a visual analog scale (VAS)], number of days with acute medication intake (n/month), and number of days elapsed since the last migraine attack (n), and aura phenotype (Table 1). Of the initial 40 patients, five were discarded based on the exclusion criteria. The enrolled 35 patients were subdivided in two groups: those reporting pure visual auras (MA, *n* = 20) and those reporting, in addition to visual symptoms, unilateral paresthesias and/or language symptoms (MA+, *n* = 15). For comparison, we recruited 19 healthy controls (HC) among healthcare professionals of comparable age and sex distribution as the patients. HC had no personal or family history of migraine or other types of primary headaches, nor any other overt medical condition.

**TABLE 1:** Clinical and demographic characteristics of healthy controls (HC), migraine with exclusively visual aura (MA) patients and migraine with complex neurological aura (MA+) patients scanned between attacks.

Characteristics	HC (*n* = 19)	MA (*n* = 20)	MA+ (*n* = 15)	Statistics
Female (*n*)	11	11	10	χ = 0.506; *p* = 0.777
Age (years)	28.4 ± 4.1	34.6 ± 10.2	28.8 ± 8.2	*p* = 0.20
Duration of migraine history (years)	–	15.5 ± 9.7	11.0 ± 6.8	*p* = 0.07
Global attack frequency/month (*n*)	–	2.9 ± 2.5	2.5 ± 2.5	*p* = 0.649
Severity of headache attacks (0–10 VAS score)	–	7.3 ± 1.6	8.0 ± 1.1	*p* = 0.139
Number of acute medication intake/month (*n*)	–	2.9 ± 2.5	1.6 ± 1.4	*p* = 0.08
Number of days elapsed since the last attack (*n*)	–	15.4 ± 16.4	22.5 ± 12.8	*p* = 0.258
Scintillating scotoma/fortification spectra	–	100%	100%	–
Sensory symptoms	–	–	100%	–
Language symptoms	–	–	26.66%	–

Data are expressed as means ± SD.

All MRI sessions were performed in the afternoon (between 4.00 and 7.00 p.m.). Participants were instructed to refrain from taking alcoholic or caffeine-containing beverages, analgesics or other medications the day before and the day of the scanning session.

All participants received a detailed description of the study and granted written informed consent. The ethical review board of the Faculty of Medicine, University of Rome, Italy, approved the project (RIF.CE 4839).

### 2.2. Data acquisition

All participants were scanned with a 3T Siemens scanner (Verio, Siemens Medical System, Erlangen, Germany) at the “Umberto I” Hospital MR Research Center, Sapienza University, Rome (Italy).

Diffusion tensor imaging images were obtained with a single-shot echo-planar image sequence with the following parameters: repetition time (TR) = 9,300 ms, echo time (TE) = 88 ms, field of view (FOV) = 192 mm × 192 mm, matrix = 96 × 96, 2 mm × 2 mm in-plane resolution, slice thickness = 2 mm, 72 continuous axial slices with no gap, one volume anterior to posterior (AP) phase of encoding *b* = 0 s/mm^2^, *b* = 1,000 s/mm^2^, 30 diffusion directions were isotropically distributed on a sphere where one direction lacked diffusion weighting resulting in 30 volumes AP phase of encoding and one volume posterior to anterior phase of encoding *b* = 0.

Structural anatomic scans were performed using T1-weighted sagittal magnetization-prepared rapid gradient echo (MP-RAGE) series [repetition time (TR) = 1,900 ms, echo time (TE) = 2.93 ms, 176 slices, 0.508 mm × 0.508 mm × 1 mm voxels].

We acquired an interleaved double-echo Turbo Spin Echo sequence proton density and T2-weighted images, and their parameters were repetition time (TR) = 3,320 ms, echo time (TE) = 10/103 ms, field of view (FOV) = 220 mm × 220 mm, matrix = 384 × 384, slice thickness = 4 mm, gap = 1.2 mm, 50 axial slices.

### 2.3. Image analysis

#### 2.3.1. Diffusion tensor imaging (DTI) and tract-based spatial statistics (TBSS)

Before pre-processing, all the DTI image volumes were visually inspected to screen noisy artifacts due to cardiac pulsations, signal dropout, and motion artifacts.

The diffusion images were processed with the Oxford Center for Functional MRI of the Brain’s (FMRIB) Software Library (FSL version 6.0)^1^ (Smith et al., 2004; Woolrich et al., 2009; Jenkinson et al., 2012).

Firstly *b* = 0 volumes AP and PA phase encoding direction were used as reference in the following FSL’s step. Topup tool estimates (Andersson et al., 2003) and corrects (Smith et al., 2004) susceptibility induced distortions. The brain extraction tool (BET) created brain masks from *b* = 0 volumes (Smith, 2002). Eddy tool performs eddy currents and movements corrections of the images.

Also quality control framework was used to assess diffusion MRI data (Bastiani et al., 2019).

The FSL toolbox DTIFIT fits the pre-processed image based on a diffusion tensor model to yield fractional anisotropy (FA), mean (MD), axial (AD), and radial (RD) diffusivity.

TBSS was used to conduct the FA voxel-wise statistical analysis according to the following steps (Smith et al., 2006): briefly, each participants’ FA image was registered to a standard space [a 1 mm isotropic FA image (FMRIB58_FA)] with the non-linear registration tool FNIRT (FMRIB’s Non-linear Registration Tool).

We did not create a study specific template by means of a tensor-based approach (Bach et al., 2014). A mean FA image was calculated based on all the participants’ images, which were then thinned to obtain the center of major WM tracts common to all subjects with an FA threshold of >0.2. To avoid misalignment during registration, each participant’s aligned FA map was projected onto the nearest relevant tract center of the mean FA skeleton by searching perpendicular to the local skeleton structure. One skeleton was created using all participants to analyze group differences. We assessed the other three DTI metrics using the same steps used to analyze the FA; the FA, MD, AD, and RD would thus show more information about the different neural mechanisms of these groups. Based on non-parametric testing (Randomize v2.9), FSL’s permutation was applied to compare the MA, MA+, and HC groups.

#### 2.3.2. Surface based morphometry (SBM)

The raw and pre-processed T1-structural images were manually inspected for artifacts and image quality.

Moreover, check sample homogeneity function in CAT12^2^ has been used to identify images with poor quality and incorrect pre-processing.

None of the acquired and pre-processed participants’ series showed abnormalities.

The CAT12 algorithms are totally automated to estimate cortical thickness.

This method segments tissues to calculate white matter distance, then it projects the local maxima, that is the cortical thickness, to other gray matter voxels by means of a neighbor relationship described by WM distance (Dahnke et al., 2013). The projection-based thickness, called PBT, handles partial volume information, sulcal blurring, and sulcal asymmetries.

This processing (Yotter et al., 2011a) involves three steps: topological correction, spherical mapping, and registration. The first step starts using the original MRI intensity values to fill or cut each topological defect. The spherical map of the uncorrected brain surface mesh is modified, and it reconstructs defective areas based on spherical harmonics low pass filter. Thereafter the spherical map, previously meshed, is reparametrized in a common coordinate system, in order to allow inter subject analysis (Yotter et al., 2011b). This software used Dartel Algorithm to the surface in order to work with spherical maps (Ashburner, 2007). The sulcal depth is reparametrized and the shape index is calculated on the sphere, by applying a multi-grid approach, to estimate a flow field that deforms spherical grid.

#### 2.3.3. Statistical analysis

We performed TBSS to conduct six t-contrasts between the three groups using age and sex as covariates. Multiple comparisons were corrected using the threshold-free cluster enhancement (TFCE) method at *p* < 0.05.

The computational Anatomy Toolbox CAT12 was used for all the statistical analyzes. A two-sample *t*-test was performed to compare the cortical thickness of patients’ subgroups to that of the control group, and between MA and MA+ subgroups of patients.

The patients’ thickness changes were assessed with a threshold of *p* < 0.01 family wise error (FWE) corrected for multiple comparison. Linear regression models were developed between the cortical thickness of each MA and MA + patients’ gray matter and clinical characteristics [duration of migraine history (years), severity of headache attacks [0–10 Visual Analog Scale (VAS) score], days from the last migraine attack (n), and monthly attacks of migraine (n)].

## 3. Results

Tract-based spatial statistics revealed no significant differences in FA, MD, RD, and AD maps between HC and the two subgroups of migraine patients.

Surface-based morphometry analysis showed significant differences in several brain areas between HC and MA and between HC and MA+, while no significant differences emerged among MA and MA+.

### 3.1. Migraine with pure visual auras (MA)

**FIGURE 1:**
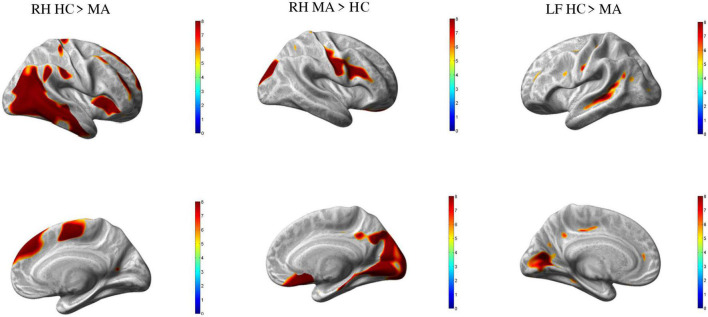
Group differences healthy controls (HC) vs. MA, *p* < 0.01 [family wise error (FWE) corrected]. Surface-based statistical maps showing clusters vertices of significant cortical thinning (LH and RH HC > MA) or thicker (RF MA > HC) in patients with pure visual auras (MA).

In comparison to HC, MA patients showed 18 clusters of significantly reduced cortical thickness including areas with most significant vertex MNI coordinates (Figure 1 and Tables 2, 4) in the following areas:

**TABLE 2:** Brain regions showing significant changes in thickness.

Comparison	Hemisphere	Size (voxels)	*P*-value	T max	Z max	Coordinate MNI	Brain lobe	Aal
HC vs. MA	L	2,624	0.000	12.14	7.5	–10, –68, 7	Occipital	Calcarine
		–	–	5.69	4.73	–19, –58, 16	–	Calcarine
		2,895	0.000	8.87	6.34	–45, –35, –2	Temporal	Middle temporal gyrus
		–	–	8.17	6.04	–46, –44, 12	–	Middle temporal gyrus
		–	–	7.03	5.49	–46, –56, 22	Parietal	Angular gyrus
		457	0.000	7.73	5.83	–48, –10, 27	Parietal	Postcentral gyrus
		400	0.000	7.24	5.6	–33, –26, 48	Parietal	Postcentral gyrus
		493	0.000	7.17	5.56	–15, –27, 38	–	Cingulum mid
		–	–	6.27	5.07	–14, –16, 43	–	Cingulum mid
		199	0.012	6.67	5.3	–15, –47, 35	–	Cingulum mid
		162	0.0028	6.4	5.15	–13, 26, –17	Frontal	Frontal sup orb
		274	0.002	6.12	4.99	–37, –37, –14	Temporal	Occipitotemporal area
		166	0.026	5.83	4.82	–30, 44, 17	Frontal	Dorsolateral prefrontal cortex
HC vs. MA	R	16,820	0.000	30.99	Inf	63, –49, 2	Temporal	Middle temporal gyrus
		–	0.000	26.85	Inf	55, –61, 9	–	Middle temporal gyrus
		–	0.000	20.35	Inf	57, –52, –10	–	Inferior temporal gyrus
		2,538	0.000	22.66	Inf	28, 27, 0	–	Insula
		–	–	6.57	5.24	36, 21, 10	–	Insula
		2,865	0.000	14.77	Inf	5, –10, 55	Frontal	Supp motor area
		–	–	13.62	Inf	8, –1, 49	–	Supp motor area
		–	–	8.98	6.38	11, –31, 68	Fronto-parietal	Paracentral lobule
		3,392	0.000	13.63	Inf	7, 45, 47	Frontal	Superior medial frontal
		–	–	8.67	6.38	11, –31, 68	Fronto-parietal	Paracentral lobule
		1,027	0.000	12.26	7.53	60, –29, 33	Parietal	Supramarginal
		958	0.000	10.51	6.97	40, –34, 63	Parietal	Postcentral gyrus
		575	0.000	9.89	6.74	37, 40, 29	Frontal	Middle frontal gyrus
		162	0.008	7.41	5.68	14, –59, 14	Occipital	Calcarine
		340	0.000	7.27	5.61	39, 20, 49	Frontal	Middle frontal gyrus
MA vs. HC	R	12,340	0.000	22.67	Inf	4, –72, 5	Occipital	Lingual
		–	–	20.67	Inf	13, –97, 19	–	Superior occipital gyrus
		–	–	13.32	7.82	–7, –78, 26	–	Cuneus
		7,085	0.000	18.42	Inf	51, –11, 24	Frontal	Rolandic operculum
		–	–	17.14	Inf	45, –10, 30	Parietal	Postcentral gyrus
		–	–	12.12	7.49	38, 6, 24	Frontal	Inferior frontal gyrus
		2,757	0.000	14.83	Inf	4, 20, –20	Frontal	Subcallosal cortex
		–	–	13.32	7.82	10, 37, –22	–	–
		–	–	9.17	6.46	3, 17, –6	–	–
		168	0.020	6.47	5.19	26, 12, 45	Frontal	Middle frontal gyrus

HC vs. MA left and right hemisphere.

• *Occipital lobe*: calcarine area (including striate and extrastriate areas) of both hemispheres;
• *Temporal lobe*: and occipitotemporal area on the left hemisphere; inferior temporal gyrus and insula on the right hemisphere; middle temporal gyrus of both hemispheres;
• *Parietal lobe*: angular gyrus on the left;
• *Frontal lobe*: frontal supraorbital area and hemisphere; supramarginal gyri on the right hemisphere; and postcentral gyrus of both hemispheres; dorsolateral prefrontal cortex of the left hemisphere; supplementary motor area, paracentral lobule, superior medial frontal cortex, middle cingulum and middle frontal gyrus of the right hemisphere.

By contrast, patients with MA had four clusters of significantly increased cortical thickness including areas with most significant vertex MNI coordinates in the right occipital lobe (lingual gyrus, superior occipital gyrus and cuneus), parietal lobe (postcentral gyrus), and in the frontal lobe (Rolandic operculum, inferior frontal gyrus, subcallosal cortex, and middle frontal gyrus).

### 3.2. Migraine with complex auras (MA+)

**FIGURE 2:**
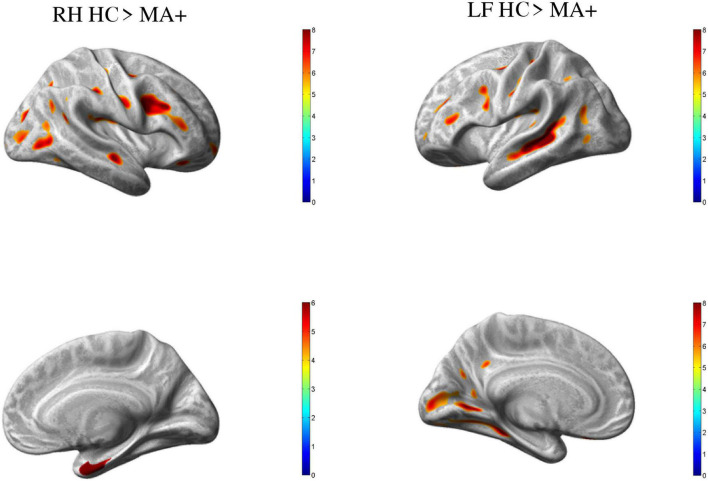
Group differences HC vs. MA, *p* < 0.01 [family wise error (few) corrected]. Surface-based statistical maps showing clusters of significant cortical thinning (LH and RH HC > MA+) or thicker (LF MA+ > HC) in patients with complex neurological auras (MA+).

Compared to HC, MA+ patients showed 30 clusters of significantly reduced cortical thickness including areas with most significant vertex MNI coordinates (Figure 2 and Tables 3, 4) in the following areas:

**TABLE 3:** HC vs. MA+ left and right hemisphere.

Comparison	Hemisphere	Size (voxels)	*P*-value	T max	Z max	Coordinate MNI	Brain lobe	Aal
HC vs. MA+	L	773	0.000	12.12	7.18	–26, –61, 35	Occipital	Middle occipital gyrus
		2,952	0.000	11.35	6.96	–45, –36, –1	Temporal	Temporal gyrus
		–	–	8.79	6.09	–46, –23, –9	–	Middle temporal gyrus
		–	–	6.99	5.31	–49, –15, –16	–	Middle temporal gyrus
		1,025	0.000	8.31	5.90	–37, –36, –15	–	Inferior temporal gyrus
		–	–	6.70	5.17	–28, –62, –5	–	–
		397	0.000	8.12	5.81	–12, –63, 5	Occipital	Calcarine
		696	0.000	8.11	5.81	–20, –63, 5	–	Calcarine
		–	–	7.61	5.60	–20, –58, 1	–	Lingual
		442	0.000	7.88	5.71	–26, –5, 46	Frontal	Supplementary motor area
		802	0.000	7.54	5.57	–36, 4, 14	Temporal	Central operculum
		353	0.000	7.27	5.44	–26, –26, 50	Frontal	Precentral gyrus
		801	0.000	7.14	5.38	–13, –89, 6	Occipital	Superior occipital gyrus
		–	–	5.76	4.55	–11, –68, 11	–	Calcarine
		549	0.000	7.08	5.35	–41, 7, 23	Frontal	Inferior frontal operculum
		–	–	6.97	5.30	–36, 10, 34	–	Middle frontal cortex
		453	0.000	7.04	5.34	–46, –12, 29	Parietal	Postcentral gyrus
		–	–	6.01	4.81	–25, –18, 38	–	–
	R	1,107	0.000	11.19	6.91	28, –75, 18	Occipital	Superior occipital gyrus
		2,639	0.000	9.54	6.37	38, 3, 24	Frontal	Precentral gyrus
		–	–	8.67	6.04	38, 22, 26	–	Inferior frontal gyrus
		–	–	6.85	5.24	37, 32, 13	–	Inferior frontal gyrus
		397	0.000	8.36	5.92	50, –18, –14	Temporal	Middle temporal gyrus
		1,121	0.000	8.35	5.91	35, –36, –15	–	Fusiform gyrus
		–	–	7.99	5.76	31, –54, –9	–	Fusiform gyrus
		–	–	7.52	5.56	22, –75, –6	Occipital	Lingual
		1,426	0.000	8.28	5.89	38, –15, 26	Frontal	Rolandic operculum
		–	–	7.12	5.37	40, –29, 22	–	–
		–	–	6.30	4.96	46, –17, 14	–	–
		536	0.000	7.91	5.73	45, –30, 35	Parietal	Supramarginal gyrus
		848	0.000	7.69	5.62	43, –63, 2	Temporal	Middle temporal gyrus
		–	–	7.65	5.62	42, –72, –8	Occipital	Inferior occipital gyrus
		–	–	6.25	4.94	42, –67, 12	–	–
		363	0.000	7.54	5.57	28, –5, 46	Frontal	Premotor cortex
		323	0.000	7.48	5.54	24, 34, 12	Temporal	Hippocampal region
		427	0.000	7.44	5.52	33, –28, 48	Parietal	Postcentral gyrus
		400	0.000	7.35	5.48	14, –16, 42	–	Cingulum mid
		628	0.000	7.26	5.44	49, 10, 24	Frontal	Inferior frontal operculum
		239	0.003	7.07	5.35	48, –53, 19	Temporal	Middle temporal gyrus
		240	0.003	6.97	5.30	19, 56, –5	Frontal	Frontal supraorbital cortex
		237	0.003	6.90	5.27	21, –62, 42	Occipital	Superior occipital gyrus
		457	0.000	6.81	5.22	32, –84, 2	Occipital	Middle occipital gyrus
		216	0.006	6.21	4.81	21, –53, 3	Occipital	Lingual

**TABLE 4:** Synoptic table of cortical areas with reduced gray matter in migraine with pure visual auras (MA) and migraine with complex visual auras (MA+) in comparisons with HC.

Regions with reduced cortical thickness		MA (R)	MA (L)	MA+ (R)	MA+ (L)
Occipital lobe	Calcarine area	X	X	–	X
	Inferior occipital gyrus	–	–	X	–
	Middle occipital gyrus	–	–	X	X
	Superior occipital gyrus	–	–	X	X
	Lingual gyrus	–	–	X	X
Temporal lobe	Occipito-temporal area	–	X	–	–
	Middle temporal gyrus	X	X	X	X
	Inferior temporal gyrus	X	–	–	X
	Central operculum	–	–	–	X
	Fusiform gyrus	–	–	X	–
	Hippocampal region	–	–	X	–
	Insula	X	–	–	–
Parietal lobe	Postcentral gyrus	X	X	X	X
	Supramarginal gyrus	X	–	X	–
	Angular gyrus	–	X	–	–
Frontal lobe	Frontal supraorbital area	–	X	X	–
	Dorsolateral prefrontal cortex	–	X	–	–
	Supplementary motor area	X	–	–	X
	Paracentral lobule	X	–	–	
	Superior medial frontal cortex	X	–	–	X
	Middle cingulum	X	–	X	
	Middle frontal gyrus	X	–		X
	Inferior frontal operculum	–	–	X	X
	Premotor cortex	–	–	X	–
	Inferior frontal gyrus	–	–	X	–
	Rolandic operculum	–	–	X	X
	Precentral gyrus	–	–	X	X

In black thinner areas in both MA and M+ subgroups, but in opposite hemispheres; in light blue thinner areas in both MA and MA+ in the same hemisphere; in red thinner areas in the MA+ subgroup only; in orange thinner areas in the MA subgroup only. R, right hemisphere; L, left hemisphere; MA, migraine with pure visual auras; MA+, migraine with complex visual auras.

• *Occipital lobe*: calcarine area of the left hemisphere; inferior occipital gyrus of the right hemisphere; lingual gyrus, middle and superior occipital gyrus of both hemispheres;
• *Temporal lobe*: inferior temporal gyrus, and central operculum of the left hemisphere, fusiform gyrus, and hippocampal region of the right hemisphere; middle temporal gyrus for both hemispheres;
• *Parietal lobe*: supramarginal gyrus on the right hemisphere; postcentral gyrus for both hemispheres;
• *Frontal lobe*: supplementary motor area, and middle frontal gyrus of the left hemisphere; inferior frontal gyrus, premotor cortex, middle cingulum, and frontal supraorbital cortex of the right hemisphere; Rolandic operculum, inferior frontal operculum, precentral gyrus for both hemispheres.

The differences and similarities between the two subgroups of migraine with aura patients, concerning SBM changes are highlighted in Table 4.

Linear regression models revealed no statistically significant relationship between morphometric and clinical variables.

## 4. Discussion

To the best of our knowledge, this is the first published study comparing brain surface-based morphometry and tract-based spatial statistics in the same migraine patients with different aura phenotypes to healthy controls. Compared to the latter, we found in patients with pure visual auras (MA) a reduced thickness of certain cortical areas of the occipital, temporal, parietal, and frontal lobes on both sides, whereas the cortex was thicker in areas of the right occipital, parietal, and frontal lobes. By contrast, only areas with reduced cortical thickness were observed in patients with complex neurological auras (MA+) bilaterally in occipital, temporal, parietal, and frontal lobes. Our results also indicate that these gray matter thickness abnormalities are not associated with an altered microstructure of white fiber bundles. This concords with Petrušić et al. (2018) study showing no statistical difference between the subgroups of migraineurs with aura and healthy subjects in the structure of the white matter fiber bundles, but only subtle abnormalities when a more liberal statistical threshold was used.

We found no significant structural differences when MA and MA+ were compared between each other. The latter findings do not agree completely with the SBM study by Petrusic et al. (2018) who report no change in cortical thickness between HC and migraine with aura patients, but increased sulci depths in left frontal and temporal lobes in MA+ compared to MA patients. In another publication, the same group reports a positive correlation between the aura complexity score and cortical thickness in several areas including primary visual cortex (Petrusic et al., 2018, 2019), while in our study the only significant increase in cortical thickness compared to HC was found in patients with pure visual auras and not in those with complex auras. These discrepancies could in part be due to methodological differences: we used a three Tesla scan instead of 1.5T in the Petrusic et al.’s study and subdivided patients in only two large groups (simple visual *n* = 20 or complex auras *n* = 15) while Petrusic et al. distinguished three subgroups with low numbers of subjects: simple auras (*n* = 14) moderately complex (*n* = 9) and complex auras (*n* = 9).

Several neuroimaging studies showed common and distinct morphofunctional characteristics between subgroups of migraine with aura patients (Sándor et al., 2005; Petrusic et al., 2019; Coppola et al., 2021; Silvestro et al., 2022). Both MA and MA+ patients had altered functional connectivity between the default mode network and the dorsal attentional system, but patients with MA+ displayed lower values of thalamic diffusivity metrics than MA patients and healthy subjects (Coppola et al., 2021). In another resting-state fMRI study, the sensorimotor network of both migraine with aura phenotypes had lower intrinsic functional connectivity of the bilateral superior temporal gyrus, left precentral gyrus, and cingulate gyrus compared to healthy subjects, while the visual network showed higher connectivity, centered on the right lingual gyrus, as compared to patients without aura and healthy subjects (Silvestro et al., 2022). They found also that functional connectivity of the left lingual gyrus and the right anterior insula with the sensorimotor network was higher in MA+ patients than in migraine without aura or with pure visual aura (Silvestro et al., 2022). In an MR spectroscopy study of the occipital cortex Sándor et al. (2005) found a progressive increase in lactate during sustained visual stimulation in MA+ patients, whereas in MA patients lactate levels were already elevated before the stimulation without further increase during the stimulation. These differences were also highlighted in an evoked potential study where visual pathway activity was greater in complex aura sufferers than in pure visual aura, but both showed a deficient habituation to visual evoked potentials (Coppola et al., 2015). In a recent electrophysiological study, latency of the P3 event-related potential obtained with a visual oddball paradigm was significantly longer in MA+ patients than in MA or HC (Petrusic et al., 2022).

Our results indicate overall that structural abnormalities are more diffuse in migraineurs with complex auras than in those with pure visual auras (see Table 4), the latter displaying a combination of increased and decreased cortical thickness. However, it is noteworthy that MA patients, despite having less numerous and disseminated areas of cortical thinning, disclose some clusters that have a greater extent than in patients with MA+. This occurs especially in temporal and visual regions, which includes the calcarine cortex and lingual gyrus. Whether this extensive cortical gray matter involvement could be the cause or the consequence of the above described functional abnormalities detected with electrophysiological and neuroimaging methods remains to be determined (Coppola et al., 2019, 2020).

### 4.1. SBM changes common to the two migraine with aura subgroups

Our analysis of whole-brain surface thickness showed a partial overlap between areas of altered gray matter thickness with areas previously found to be functionally abnormal in migraine with aura. For instance, both MA and MA+ patients had a larger surface thickness reduction compared to controls in clusters comprising left calcarine area–including striate and extrastriate areas –, bilateral middle temporal gyri, bilateral postcentral gyri (S1) and right supramarginal gyrus, all areas participating to some degree in the processing of various pain-related cognitive aspects. This concords with previous studies reporting deactivation of extensive occipital, frontal and temporal cortical areas during central sensitization (Iannetti et al., 2005) that is known to be one of the mechanisms underlying hyperalgesia and allodynia during the migraine headache phase (Coppola et al., 2013). In particular, the large calcarine area is involved in the processing of cognitive, affective, and sensory aspects of pain (Coghill et al., 1999; Coppola et al., 2010), the postcentral gyri in that of pain-related emotions and upcoming pain prediction (Yoshino et al., 2017; Koppel et al., 2022) in conjunction with the middle cingulate cortex and the insula (both thinned in MA). The insula, in turn, is a hub highly connected with areas of the temporal and frontal lobes, the latter showing thinned cortical areas both in MA and MA+. Another cortical area that we found equally reduced in thickness in both subgroups of patients is the right supramarginal gyrus. In the right hemisphere this area has previously been implicated in the recognition of others’ emotions (Wada et al., 2021), including those related to pain perception (Zhao et al., 2021), while it is commonly considered essential for visuo-spatial awareness, in conjunction with the angular gyrus and inferior parietal lobe, being part of the circuitry involved in the hallucinatory experience of dreaming (Pace-Schott, 2005).

### 4.2. SBM changes specific to a migraine with aura subgroup

We also found specific thickness changes in the two subgroups. The lingual gyrus was found to be thicker in the right hemisphere of MA patients, while it was thinner bilaterally in MA+ patients, together with the adjacent middle and superior occipital gyri, These results are in line with those showing functional abnormalities in the lingual gyrus and adjacent occipital areas proportional to the aura complexity (Petrusic et al., 2019; Coppola et al., 2021; Silvestro et al., 2022). It must be noted, however, that, besides its involvement in the pathophysiology of the migraine aura (Tedeschi et al., 2016), and other abnormal visual perceptions (Schankin et al., 2014), the lingual gyrus may also be pathophysiologically implicated in migraine without aura (Zhang et al., 2020).

In our study only MA patients had an increased cortical thickness of areas belonging to the sensorimotor network (Uddin et al., 2019; Patra et al., 2021), such as the postcentral, inferior and middle frontal gyri, subcallosal cortex, and the Rolandic operculum of the right hemisphere. Of the aforementioned areas of the sensorimotor network, MA+ patients had in contrast decreased thickness only in the Rolandic operculum bilaterally. As for the lingual gyrus, it is of note that patients with complex auras have a more pronounced microstructural involvement of areas adjacent to the Rolandic operculum, such as the bilateral precentral gyrus, than those with simple visual aura.

The sensorimotor network is involved in performing/coordinating a motor task but also in a larger between-networks interaction devoted to simultaneous detection and selection of salient multisensory responses (Huang et al., 2015). That the sensorimotor network is functionally involved in both MA and MA+ was shown by Silvestro et al. (2022), with reduced functional connectivity in MA, and increased functional connectivity with the insula and lingual gyrus in MA+.

The bilateral Rolandic operculum is physiologically interconnected with the precentral gyrus including the inferior part of the central sulcus, and is one of the major regions involved in language processing (Triarhou, 2021). The left operculum is chiefly involved in sentence-level and phrase-level syntactic encoding during speech (Indefrey et al., 2001). We speculate that the bi-hemispheric involvement of the Rolandic operculum in MA+ patients may increase susceptibility to transient dysfunction of the sensorimotor and language systems, while the increased thickness of the Rolandic operculum with the other areas of the sensorimotor network of the right hemisphere in patients with MA may be more related to psychopathological features, as shown in post-stroke patients (Sutoko et al., 2020) and in social anxiety disorders (Brühl et al., 2014). The neuropathological mechanisms underlying the cortical thickness changes, especially in the language areas and the lingual gyrus, in subgroups of migraine with aura patients is not well-understood and should be further investigated in future studies.

Finally, three areas (angular gyrus, occipitotemporal area, and dorsolateral prefrontal cortex) are reduced in thickness only in pure MA and not in MA+. These areas are part of the so-called dorsal attentional system (DAS) (Corbetta and Shulman, 2002; Vossel et al., 2014; Zimmermann et al., 2018), which in migraine with aura, irrespective of the aura phenotype, had less intrinsic functional connectivity and extrinsic interconnectivity with other functional brain networks, such as the default-mode network, the executive control network, and the salience network (Niddam et al., 2016; Veréb et al., 2020; Coppola et al., 2021, 2022). The DAS is an externally oriented network that is mainly devoted to the selection and processing of relevant multisensorial, but preferentially visual, stimuli, with an additional role in response preparation (Corbetta and Shulman, 2002). The possible explanations for our findings remain speculative. It is conceivable nevertheless that the microstructural abnormalities in areas belonging to the DAS, and their previously described abnormal connectivity, may be the morphofunctional counterparts for the reduced propensity of CSD waves to progress postero-anteriorly in patients in whom the aura remains exclusively confined to the visual system, a hypothesis that must be tested in a properly designed study.

#### 4.2.1. Limitations

We acknowledge several limitations of our study. First, even though the results underwent stringent statistical methods, lack of power due to the relatively small sample size may have caused bias. Second, we did not assess whether the cortical thickness abnormalities were associated with deficits in specific cognitive domains, which should be recommended in future studies. Third, we did not collect data on frequency and duration of auras that might have influenced the structural changes, although this is unlikely since we found no correlations between cortical thickness changes and clinical variables, such as duration of disease or frequency of attacks.

## 5. Conclusion

In conclusion, migraine patients with aura, irrespective of the aura phenotype, do not have abnormalities of white-matter fiber tracts, but they have gray matter alterations in multiple brain regions involved in different aspects of pain processing and multisensory integration. In patients with complex auras we observed altered thickness of a high number of disseminated small cortical area clusters, while these were larger and more focal in migraine with pure visual auras. Whether the more widespread clusters of structural abnormalities in MA+ could be related to the propensity for spreading depression to spread in these patients beyond the visual cortex to sensory and language areas remains to be determined. However, this hypothesis is supported by imaging studies showing that, as compared to simple visual auras, complex auras are accompanied by more extensive cortical hypoperfusion involving several adjacent vascular territories (Floery et al., 2012; Förster et al., 2014; Wolf et al., 2018). More studies are needed to determine whether the observed cortical abnormalities are a primary inherited anatomical predisposition to the disease, which is not excluded given that similar abnormalities have been found in pediatric patients (Guarnera et al., 2021)–or if they are a consequence of the repeated auras, which is not supported by the lack of correlation with attack frequency. Additional studies should also verify whether individual aura symptoms are to be related to focal abnormalities in the microstructure of certain brain areas such as the lingual gyrus and the Rolandic operculum.

## Data availability statement

The original contributions presented in this study are included in the article/supplementary material, further inquiries can be directed to the corresponding author.

## Ethics statement

The studies involving human participants were reviewed and approved by the Ethical Review Board of the Faculty of Medicine, University of Rome, Italy. The patients/participants provided their written informed consent to participate in this study.

## Author contributions

GC, VD, and FCar contributed to the conception and design of the study. GG, MT, MS, MF, and VD organized the database. AD and ET performed the statistical analysis. CA, GS, and FCas wrote the first draft of the manuscript. GC and JS revised the final version of the manuscript. All authors contributed to the manuscript revision, read, and approved the submitted version.

## Footnotes

1
https://fsl.fmrib.ox.ac.uk/fsl

2
http://www.neuro.uni-jena.de
